# Stress‐induced premature senescence activated by the *SENEX* gene mediates apoptosis resistance of diffuse large B‐cell lymphoma via promoting immunosuppressive cells and cytokines

**DOI:** 10.1002/iid3.356

**Published:** 2020-10-04

**Authors:** Jiyu Wang, Qianshan Tao, Ying Pan, Zhixiang Wanyan, Fengfeng Zhu, Xuanxuan Xu, Huiping Wang, Liuying Yi, Mei Zhou, Zhimin Zhai

**Affiliations:** ^1^ Department of Hematology The Second Affiliated Hospital of Anhui Medical University Hefei Anhui China; ^2^ Department of Hematology, Jingzhou Hospital, Tongji Medical College Huazhong University of Science and Technology Wuhan Hubei China

**Keywords:** DLBCL, immunosuppressive cells, SASP, *SENEX* gene, stress‐induced premature senescence

## Abstract

**Background:**

The underlying cause of relapsed and refractory (r/r) diffuse large B‐cell lymphoma (DLBCL) is usually related to apoptosis resistance to antitumor drugs. The recent years have provided lots of evidence that tumor cells may undergo stress‐induced premature senescence (SIPS) in response to chemotherapy, but how SIPS affects lymphoma cells remains inconclusive.

**Methods:**

Fifty‐two DLBCL patients, including 6 newly diagnosed (ND), 17 complete remissions (CR), and 29 (r/r), were enrolled in this study. We used a senescence‐associated‐β‐galactosidase (SA‐β‐Gal) staining kit for senescence staining. Suppressive immune cells including regulatory T cells (Treg) and myeloid‐derived suppressor cells (MDSC) were detected by flow cytometry (FCM). Secreted cytokines were measured by ELISA Kit and *SENEX* gene expression was detected by a quantitative real‐time polymerase chain reaction. We used 40 nM doxorubicin to induce the SIPS model of DLBCL in vitro. Apoptosis and proliferation activity of senescent LY8 cells were respectively detected by FCM and CCK8. *SENEX* gene was silenced by RNA interference.

**Results:**

The proportion of senescent lymphoma cells was significantly increased in r/r DLBCL patients, concomitant with increased Treg, MDSC, and various secreted cytokines with proinflammatory and immunosuppressive effects. The *SENEX* gene was significantly elevated in the SIPS model. Senescent DLBCL cells had good antiapoptotic ability and proliferative activity accompanied by increased immunosuppressive cytokines. Interestingly, when we silenced the *SENEX* gene in the DLBCL cell line, the results were the opposite to the above.

**Conclusion:**

SIPS activated by the *SENEX* gene mediates apoptosis resistance of r/r DLBCL via promoting immunosuppressive cells and cytokines.

## Graphical abstract

We propose premature senescence induced by the SENEX gene mediates apoptosis resistance of large B‐cell lymphoma (DLBCL) via promoting immunosuppressive cells and cytokines.

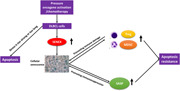

**Abbreviations**
CR: complete remission
DLBCL: diffuse large B‐cell lymphoma
FCM: flow cytometry
MDSC: myeloid‐derived suppressor cells
ND: newly diagnosed
PBMCs: peripheral blood mononuclear cells
r/r: relapse and refractory
SASP: senescence‐associated secretory phenotype
SA‐β‐Gal: senescence‐associated‐β‐Galactosidase
SIPS: stress‐induced premature senescence
Treg: regulatory T cells

## 1 INTRODUCTION

Diffuse large B‐cell lymphoma (DLBCL) is the most common type of adult aggressive lymphoma and is highly heterogeneous in clinical manifestation and prognosis.^1^ Though Rituximab based immunotherapy have been applied for several years, the clinical outcome of DLBCL patients remains challenging, as about 30%−40% of patients relapse, and 10% of them are refractory.^2^ Even with high‐dose chemotherapy combined with autologous hematopoietic stem cell transplantation (ASCT) or chimeric antigen receptor T (CAR‐T) therapy, the prognosis of some relapsed and refractory (r/r) DLBCL patients is still not optimistic.^3^, ^4^ Further understanding the underlying cause and pathogenesis of r/r DLBCL will bring about new hope for future treatment.

Cell senescence is a stable cell‐cycle arrest state. It is a fail‐safe program initiated by the body in response to severe cell damage (such as oncogene activation or DNA damage caused by chemotherapy), which induces damaged cells to enter the state of senescence to prevent potentially harmful cells from further expansion by initiating gene reprogramming.^5^, ^6^ It is usually divided into replicative senescence (RS) and stress‐induced premature senescence (SIPS) according to different mechanisms.^5^, ^6^, ^7^, ^8^ SIPS is telomere independent and may occur with internal carcinogen activation, or external drugs, oxidation, infection, ion radiation, and other DNA damage stimuli.^7^ When the pressure is removed or the environment changes, it may reenter the cell cycle and re‐start proliferation.^9^

Due to its characteristic of limiting excessive or aberrant cellular proliferation, SIPS was initially identified as a tumor‐suppression mechanism and played a key role in preventing the development of tumors.^9^ However, what cannot be understood is that some progeroid syndromes show a high incidence of tumors.^10^ In addition, the last two decades have provided mounting evidence that senescent cells are causatively involved in tumor progression.^6^, ^9^

SIPS' contribution to tumor progression also includes the formation of an immunosuppressive microenvironment.^11^ In particular, senescent cells undergoing stress are characterized by the senescence‐associated secretory phenotype (SASP),^12^ which refers to the excessive production of various cytokines, chemokines, growth factors, extracellular matrix components and remodeling proteins.^12^ Importantly, the composition of the SASP also participates in various steps of tumor progression.

The genes that regulate cellular senescence are extremely complicated. Recently, a novel gene *SENEX* has been proved to be related to cellular senescence and provides a unique gatekeeper function in the SIPS pathways in ECs.^13^ Besides this, the *SENEX* gene also involves in regulating tumor cell growth and metastasis.^14^, ^15^ Our previous research suggested that the *SENEX* protein was significantly increased in senescent DLBCL cells.^16^ However, the role of the *SENEX* gene and *SENEX* activated SIPS in DLBCL, especially in r/r DLBCL, and how SIPS affects r/r DLBCL has not been previously investigated. Here, we demonstrate that *SENEX* gene activated SIPS mediates apoptosis resistance of lymphoma cells in relapsed/refractory DLBCL (r/r DLBCL).

## 2 MATERIALS AND METHODS

### 2.1 Patients

Fifty‐two patients diagnosed with DLBCL from April 2017 to April 2019 at the Second Hospital of Anhui Medical University, with no congenital/acquired immunodeficiency, were enrolled. According to the Chinese guidelines for diagnosis and treatment of DLBCL (2013),^17^ the patients were diagnosed and divided into the newly diagnosed (ND) group, complete remission (CR) group, and r/r group. The detailed clinical data of the patients are shown in Table 1. This study was approved by the Institutional Review Board (IRB) Institutional of the Second Hospital of Anhui Medical University. All patients enrolled in the study signed informed consent.

**Table 1:** Characteristics of DLBCL patients

State of disease at sample draw	Number of patients	Average age (range)	Gender distribution
Newly diagnosed	6	59.3 (47−79)	Male 4
			Female 2
Complete response	17	50.4 (31−76)	Male 13
			Female 4
Relapsed/refractory	29	60.3 (46−85)	Male 24
			Female 5

Abbreviation: DLBCL, diffuse large B‐cell lymphoma.

### 2.2 Cell culture

The human DLBCL cell line, OCI‐LY8 (abbreviation in this article: LY8) was cultured in RPMI‐1640 supplemented with 10% fetal bovine serum. Cell cultures were maintained and incubated at 37°C in humidified air with 5% CO_2_.

### 2.3 Induction of senescence

Doxorubicin was purchased from Energy Chemical as a 10 mg/bottle lyophilized powder. The doxorubicin lyophilized powder was diluted in RPMI‐1640. Peripheral blood mononuclear cells (PBMCs) (10^6^/ml) extracted from DLBCL patients with lymphoma cell infiltration in peripheral blood and LY8 cells (10^6^/ml) were treated with 10, 20, and 40 nM doxorubicin, respectively, every 2 days for 2 h each for a total of six induction times in vitro.

### 2.4 Senescence staining

According to instructions for the Senescence‐associated‐β‐Galactosidase (SA‐β‐Gal) Staining Kit (Beyotime), LY8 cells treated with 10, 20, and 40 nM doxorubicin respectively were fixed with galactosidase fixative and incubated in dyeing working fluid as previously described.^16^ Finally, the stained cells were observed under a microscope (CNOPTEC). Cells that stained green‐blue were evaluated as positive senescent cells. All SA‐β‐gal assays reflect at least three samples.

### 2.5 Flow cytometry

Cellular phenotype analysis and apoptosis analysis were detected by a flow cytometer FC‐500 (Beckman Coulter). Regulatory T cells (Treg) were subjected to staining and identified by CD4^+^CD25^+^CD127^low^ (Beckman Coulter). Granulocytic myeloid‐derived suppressor cells MDSC subsets (G‐MDSC) were subjected to staining and identified by CD11b^+^CD33^+^HLA‐DR^–^CD14^–^CD15^+^CD66b^+^, and monocytic MDSC subsets (M‐MDSC) were subjected to staining and identified by CD11b^+^CD33^+^HLA‐DR^‐^CD14^+^CD15^–^CD66b^–^ (Beckman Coulter). LY8 cells were treated with/without doxorubicin in vitro and then stained with annexin V and propidium iodide (Biouniquer) for apoptosis analysis according to the manufacturer's instructions.

### 2.6 Western blot

Total proteins from cells were extracted by western blot with IP cell lysis liquid (Beyotime) according to standard procedures. Primary antibodies used for western blot are shown in Table 2. Proteins were analyzed using the SuperSignal West Femto Trial Kit (Thermo Fisher Scientific) as previously described.^18^

**Table 2:** Primary antibodies used for western blot

Name	Company	Item number	Dilution ratio
Anti‐ARHGAP18	Abcam	ab175970	1:1000
P16 INK4A(D7C1M) Rabbit mAb	Cell Signaling Technology	#80772	1:1000
Rb(D20) Rabbit mAb	Cell Signaling Technology	#9313	1:1000
Phospho‐Rb(Ser780)(D59B7) Rabbit mAb	Cell Signaling Technology	#8180	1:1000
Mouse anti‐β‐Actin mAb	ZSGB‐BIO	TA‐09	1:2000

### 2.7 Quantitative real‐time polymerase chain reaction (qRT‐PCR) analysis

Peripheral blood was collected from patients for evaluation of *SENEX* gene levels via qRT‐PCR. Total RNA in peripheral blood or LY8 cells was extracted by TRIzol (Invitrogen). RNA was reverse‐transcribed using a Transcript RT Kit (Sangon) according to the manufacturer's protocol. RT‐PCR was performed on the ABI 7500 Real‐Time PCR System (Life Technologies) by SYBR Green PCR Master Mix (TaKaRa). All primers were synthesized by Sangon. The relative *SENEX* expression level was calculated using the $2^{‐∆∆C_{t}}$ method. Sequences used for qRT‐PCR primers are shown in Table 3.

**Table 3:** Sequences used for qRT‐PCR primers

Name	Sequences (5′–3′)
GAPDH: Forward	GTGAAGGTCGGTGTGAACGG
GAPDH: Reverse	GATGCAGGGATGATGTTCTG
*SENEX*: Forward	TTGCTCTGTTTTCCAGATTGGA
*SENEX*: Reverse	GCCCCAGTGCTTGAGGCT

Abbreviations: GAPDH, glyceraldehyde 3‐phosphate dehydrogenase; qRT‐PCR, quantitative real‐time polymerase chain reaction.

### 2.8 Small interfering RNA (SiRNA) synthesis and transfection

The individual small interfering RNA target *SENEX* gene (*SENEX*‐siRNA) and scramble negative control siRNA (NC) were synthesized by Sangon. Ly8 cells were transfected with *SENEX*‐SiRNA or NC for 48 h by using lipofectamine 2000 (Invitrogen) Sequences used for siRNA transfection have been previously published.^16^

### 2.9 Proliferation analysis

LY8 cells treated with 10, 20, and 40 nM doxorubicin, respectively,  six times were plated at a density of 5000 cells/well. Cell proliferation was measured with a CCK‐8 Kit (BestBio). Each assay was performed with five replicates in three independent experiments.

### 2.10 Enzyme linked immunosorbent assay (ELISA)

Serum from DLBCL patients with lymphoma cell infiltration in peripheral blood and cell suspension from doxorubicin‐induced LY8 cells were stored at −20°C. We avoided repeated freeze‐thaw cycles. The concentration of interleukin‐2 (IL‐2), IL‐6, IL‐8, IL‐10, IL‐35, transforming growth factor‐β1 (TGF‐β1), tumor necrosis factor‐α (TNF‐α), E2F1, and vascular endothelial growth factor (VEGF) were measured with Human ELISA Kit (OmnimAbs). We used the stock solution (8000 pg/ml) to produce a twofold dilution series (including 4000, 2000, 1000, 500, and 250 pg/ml). Each group was made in triplicate. All standards and samples were added in duplicate to the microtiter plate according to standard procedures. And taking the blank well as zero, we measured the optical density at 450 nm after adding stop solution within 15 min.

### 2.11 Statistical analysis

Student's *t* test was employed for analysis of two‐sample and two‐tailed comparisons by SPSS 16.0 (SPSS Inc.). Pearson correlation was used to measure the degree of dependence between variables with SPSS 16.0. *p* values were calculated, and *p* < .05 was considered statistically significant.

## 3 RESULTS

### 3.1 Senescent lymphoma cells were increased in r/r DLBCL patients

**Figure 1:**
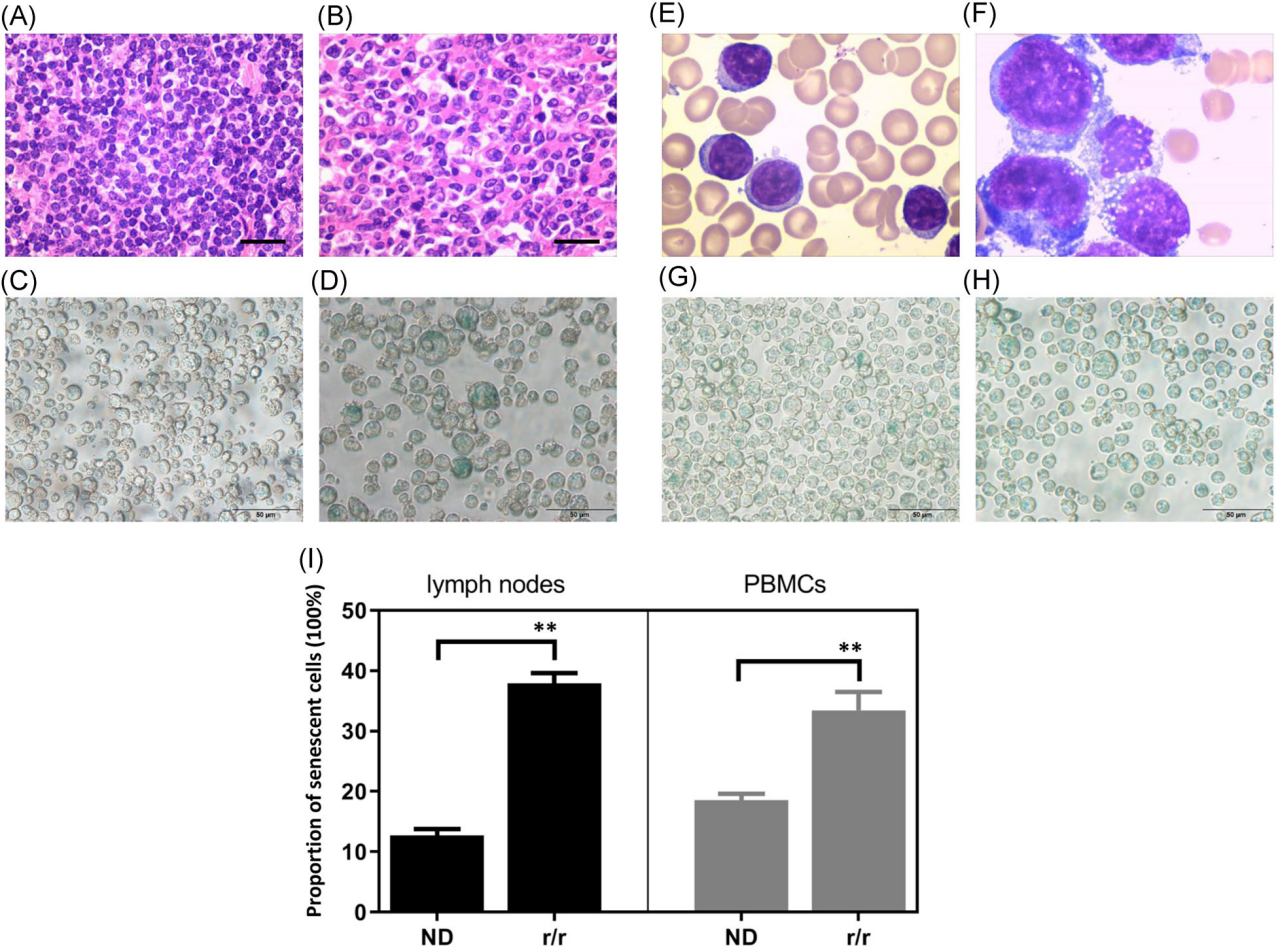
Increased senescent lymphoma cells in relapsed/refractory (r/r) diffuse large B‐cell lymphoma (DLBCL) patients. (A) Hematoxylin and eosin (HE) staining of lymph node tissue in newly diagnosed (ND) DLBCL patients, ×400, scale bar = 50 μm. (B) HE staining of lymph node tissue in r/r DLBCL patients, ×400, scale bar = 50 μm. (C) Senescence staining of lymph node tissue in ND DLBCL patients, ×400, scale bar = 50 μm. (D) Senescence staining of lymph node tissue in r/r DLBCL patients, ×400, scale bar = 50 μm. (E) Normal‐sized lymphoma cells in peripheral blood of ND patients, ×400, scale bar = 50 μm. (F) Lymphoma cells, normal lymphocyte, and even interstitial cells were significantly increased in r/r patients than other normal size lymphoma cells, ×400, scale bar = 50 μm. (G) Senescence staining of peripheral blood mononuclear cells (PBMCs) in ND patients, ×400, scale bar = 50 μm. (H) Senescence staining of PBMCs in r/r DLBCL patients, ×400, scale bar = 50 μm. (I) The proportion of senescent lymphoma cells in DLBCL patients

We performed morphological observation and senescence staining in lymph node tissue and peripheral blood specimens of DLBCL patients with peripheral blood invasion. In the hematoxylin and eosin staining of lymph nodes, we observed that the size of some lymphoma cells was increased in r/r DLBCL patients, exceeding the common large B‐cell size in ND DLBCL patients (Figure 1A,B). Then, we performed the SA‐β‐Gal activity analysis in lymph node specimens of ND and r/r DLBCL. It was found that the SA‐β‐Gal activity of enlarged lymphoma cells in r/r patients was significantly increased compared with ND patients (Figure 1C,D). Next, we performed morphological observation and senescence staining in peripheral blood specimens from ND and r/r DLBCL patients with peripheral blood invasion. The volume of abnormal lymphoma cells from r/r DLBCL patients was greater than that from ND patients (Figure 1E,F). Besides this, more SA‐β‐Gal staining positive lymphoma cells were found in peripheral blood of r/r DLBCL patients compared with ND patients (Figure 1G–I). These results suggested that the proportion of senescent cells was significantly increased in patients with r/r DLBCL.

### 3.2 Immunosuppressive cells and secreted cytokines were increased in r/r DLBCL

**Figure 2:**
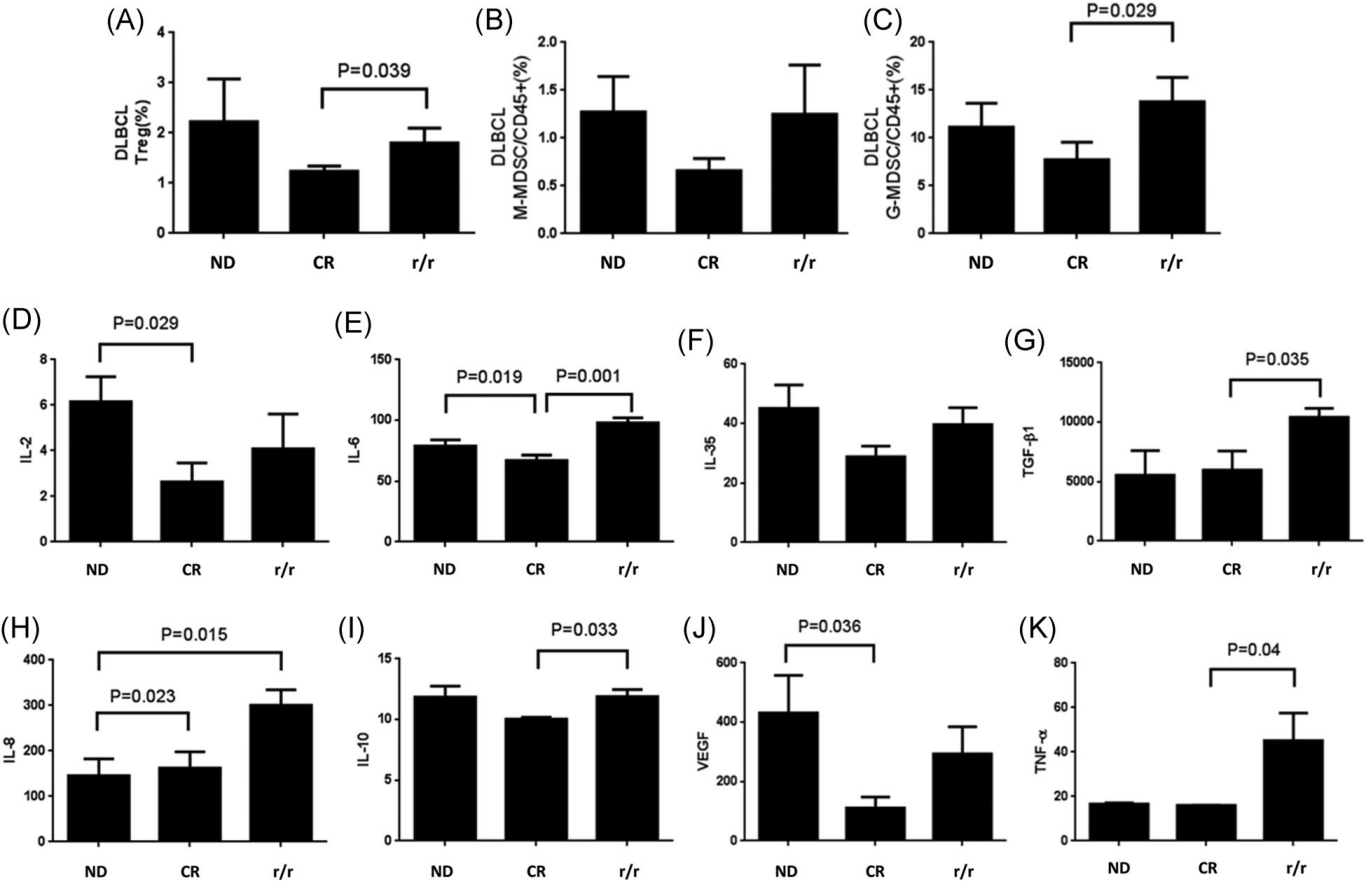
Upregulated immunosuppressive cells and cytokines in r/r DLBCL patients. (A) The level of Tregs in DLBCL patients. (B) The level of M‐MDSC in DLBCL patients. (C) The level of G‐MDSC in DLBCL patients. (D) The concentration of IL‐2 in DLBCL patients. (E) The concentration of IL‐6 in DLBCL patients. (F) The concentration of IL‐35 in DLBCL patients. (G) The concentration of TGF‐β1 in DLBCL patients. (H) The concentration of IL‐8 in DLBCL patients. (I) The concentration of IL‐10 in DLBCL patients. (J) The concentration of VEGF in DLBCL patients. (K) The concentration of TNF‐α in DLBCL patients. CR, complete remission patients; DLBCL, diffuse large B‐cell lymphoma; IL, interleukin; G‐MDSC, granulocytic myeloid‐derived suppressor cells; M‐MDSC, monocytic myeloid‐derived suppressor cells; ND, newly diagnosed patients; r/r, relapsed/refractory patients; TGF‐β1, transforming growth factor‐β1; Treg, regulatory T cells; VEGF, vascular endothelial growth factor

To analyze the tumor immune microenvironment in the peripheral circulation of patients with r/r DLBCL, we analyzed the proportion of circulated Treg and MDSC. Our results showed that the Treg percentage in the r/r group was significantly higher than that of the CR group (*p* = .039) (Figure 2A). Meanwhile, the percentage of G‐MDSC and M‐MDSC in the r/r group were both higher than that of the CR group (Figure 2B). In addition, the percentage of G‐MDSC had a statistical difference (*p* = .029) (Figure 1C). These results suggested that the peripheral circulated tumor immune microenvironment of r/r DLBCL patients under the state of immunosuppressed. The accumulation of immunosuppressive cells might be one of the important reasons that caused lymphoma cells' immune escape and drug resistance.

Senescent cells can continue to maintain metabolism and secrete various cytokines. This secretion function is called “senescence‐related secretory phenotype (SASP)”, which is one of the important mechanisms involved in the senescence process. In our study, the peripheral blood serum of DLBCL patients was collected and analyzed for the relevant secreted cytokines by ELISA. The results showed that IL‐2, IL‐6, IL‐8, IL‐35, IL‐10, TGF‐β1, VEGF, and TNF‐α expression levels in the r/r group were all increased in varying degrees than those of the CR group, and IL‐6, IL‐8, TGF‐β1, and TNF‐α expression levels were even higher than those of the ND group (Figure 2D–K). It was suggested that these increased secreted cytokines might interact and participate in the formation of a complex tumor immune microenvironment in the peripheral blood of r/r DLBCL patients, contributing to lymphoma cell proliferation, invasion, and resistance to chemoradiotherapy.

### 3.3 *SENEX* gene activation in SIPS model induced by doxorubicin

**Figure 3:**
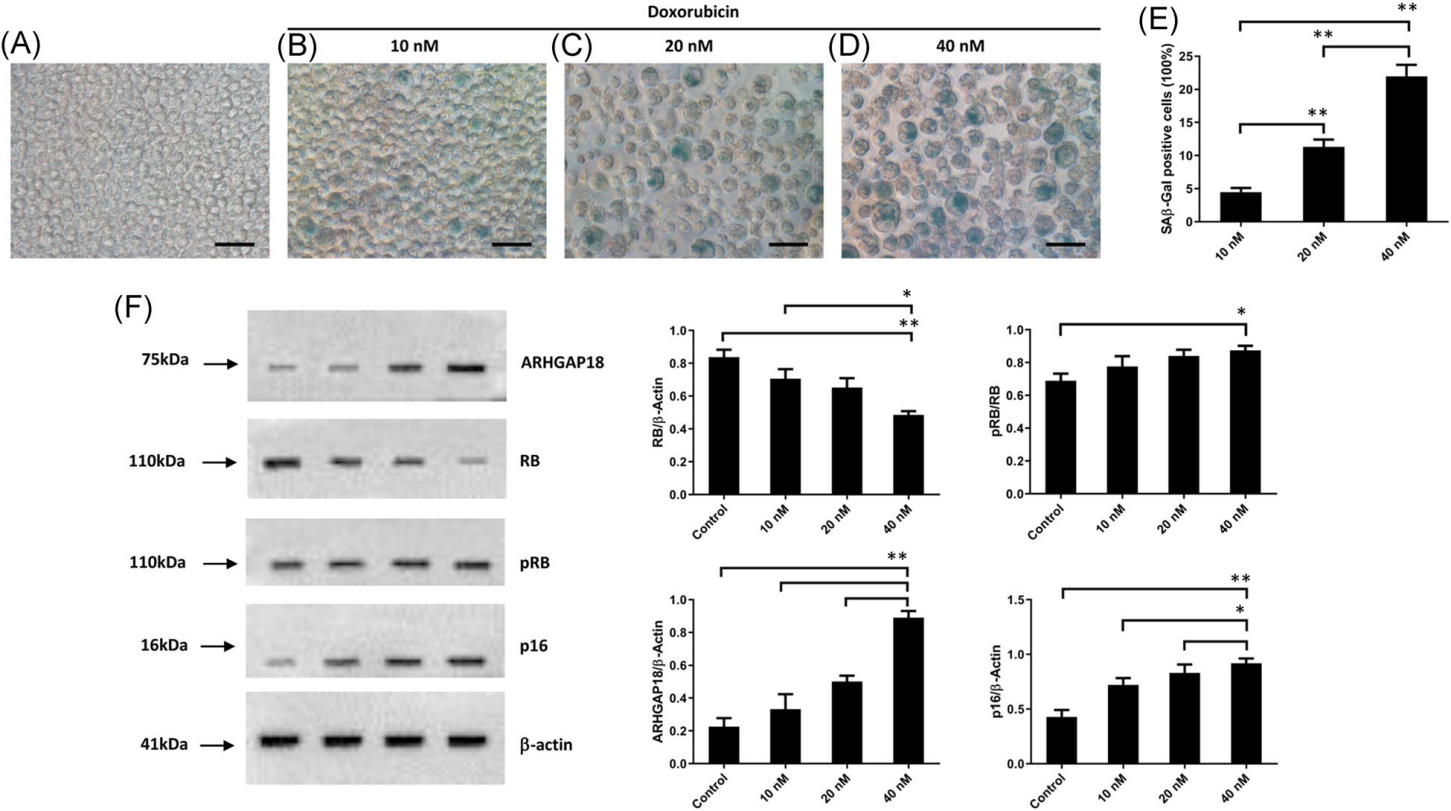
*SENEX* gene activation in the SIPS model of DLBCL induced by doxorubicin. (A) Unprocessed LY8 cells were stained for SAβ‐gal. (B–D) LY8 cells were repeatedly induced by 10, 20, and 40 nM doxorubicin, respectively, six times, 2 h each time, and then stained for β‐galactosidase. (E) The proportion of SAβ‐gal positive LY8 cells treated with different concentrations of doxorubicin. **p < .01, *p < .05. (F) LY8 cells were repeatedly induced by 10, 20, and 40 nM doxorubicin, respectively, six times, 2 h each time. And cells were harvested for western blot. The expression of ARHGAP18 and p16/RB were measured with western blot. **p< .01, *p< .05. DLBCL, diffuse large B‐cell lymphoma; SAβ‐gal, senescence‐associated‐β‐Galactosidase; SIPS, stress‐induced premature senescence

To obtain a senescence model of lymphoma cells under the stress of chemotherapy, we used different concentrations of doxorubicin (10, 20, and 40 nM) to treat the human DLBCL cell line LY8 in vitro. After repeated induction for six times, the senescence of LY8 cells was detected by SA‐β‐Gal staining. Compared with the control group, a large number of enlarged senescent cells could be detected in all the doxorubicin‐induced groups (Figure 3A–D). Interestingly, the proportion of senescent cells was increased with the increase of doxorubicin concentrations, and the highest proportion was 20.7% in 40 nM doxorubicin group (Figure 3E). Consistent with the staining results, we simultaneously tested the expression levels of the classic senescent marker p16 protein and pRB protein. We found that p16 and pRB expression levels were both significantly increased after the induction of doxorubicin, while RB expression level was decreased (Figure 3F). These results suggested that repeated use of 40nM doxorubicin in vitro could successfully induce lymphoma cells into senescent state and the p16/RB pathway was activated in senescent lymphoma cells. In addition, we also found that the expression of ARHGAP18 (the protein encoded by the SENEX gene) was significantly increased in senescent lymphoma cells (Figure 3F). These results indicated that *SENEX* expression was significantly increased in senescent DLBCL cells accompanying p16/Rb pathway activation. The overexpressed *SENEX* gene may be closely related to the premature senescence of DLBCL cells.

### 3.4 Senescent DLBCL cells promoted apoptosis resistance

**Figure 4:**
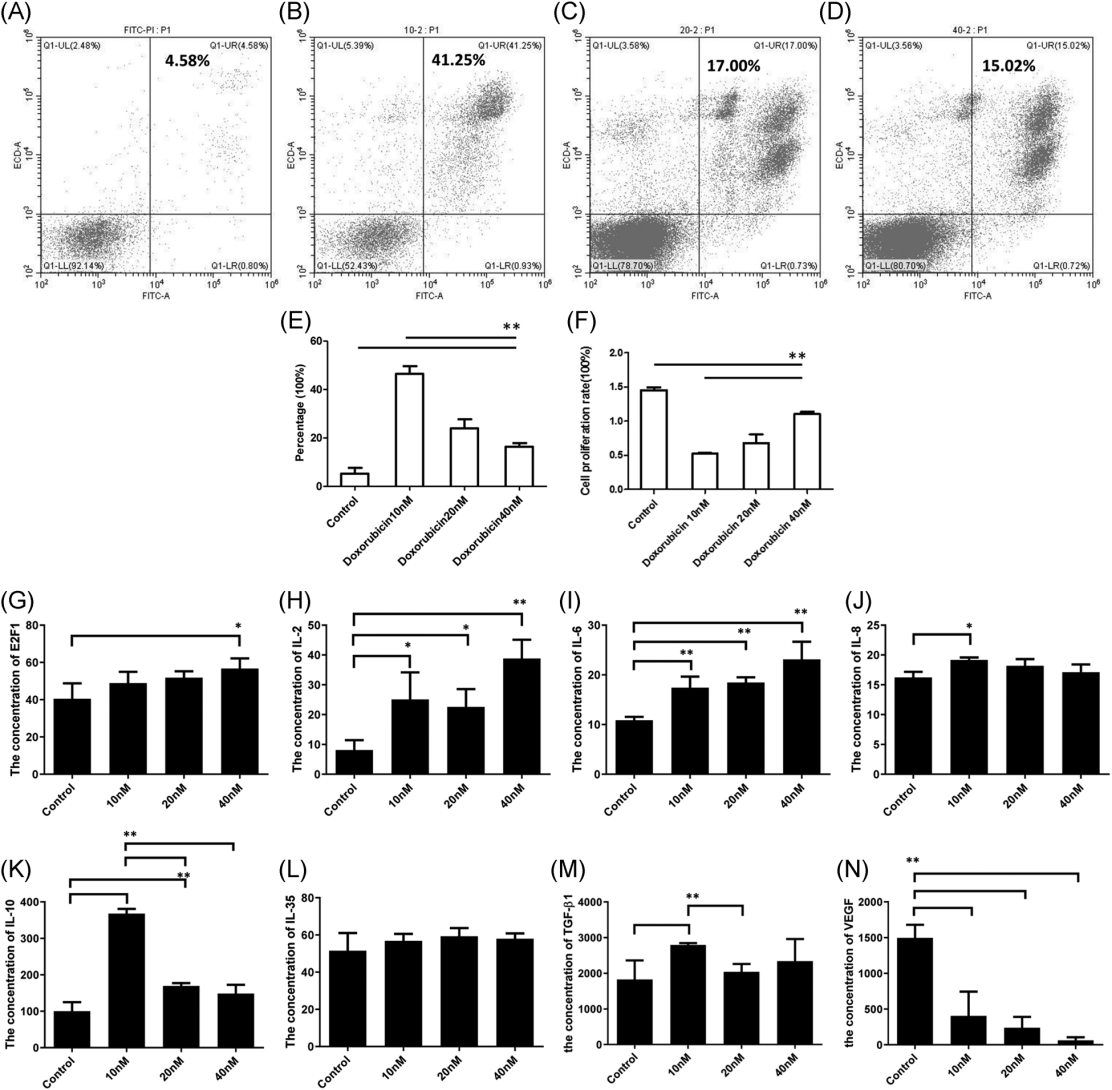
Senescent DLBCL cells promoted apoptosis resistance via increasing SASP. (A) The proportion of apoptotic cells in the control group was measured with FCM analysis (unprocessed LY8 cells). (B) The proportion of apoptotic cells in the 10 nM‐doxorubicin group was measured with FCM analysis. (C) The proportion of apoptotic cells in the 20 nM‐doxorubicin group was measured with FCM analysis. (D) The proportion of apoptotic cells in the 10 nM‐doxorubicin group was measured with FCM analysis. (E) Effect of doxorubicin‐induced SIPS on the apoptosis of LY8 cells. **p < .01 (F) Cell proliferation rate of doxorubicin‐induced LY8 cells was measured with CCK8 proliferation analysis. **p< .01 (G) The concentration of E2F1 in cell suspension of doxorubicin‐induced LY8 cells. (H) The concentration of IL‐2 in cell suspension of doxorubicin‐induced LY8 cells. (I) The concentration of IL‐6 in cell suspension of doxorubicin‐induced LY8 cells. (J) The concentration of IL‐8 in cell suspension of doxorubicin‐induced LY8 cells. (K) The concentration of IL‐10 in cell suspension of doxorubicin‐induced LY8 cells. (L) The concentration of IL‐35 in cell suspension of doxorubicin‐induced LY8 cells. (M) The concentration of TGF‐β1 in cell suspension of doxorubicin‐induced LY8 cells. (N) The concentration of VEGF in cell suspension of doxorubicin‐induced LY8 cells. (K) The concentration of TNF‐α in cell suspension of doxorubicin‐induced LY8 cells. (*n* = 3). CCK8, cell counting kit‐8; DLBCL, diffuse large B‐cell lymphoma; FCM, flow cytometry; IL, interleukin 6; SASP, senescence‐associated secretory phenotype; SIPS, stress‐induced premature senescence; TGF‐β1, transforming growth factor‐β1; TNF‐α, tumor necrosis factor‐α

To verify the biological function of senescent lymphoma cells, we next examined the apoptosis rate and proliferation activity in the senescent LY8 cells. We found that the apoptosis rate of the three doxorubicin‐induced groups were all significantly higher than that of the control group (Figure 4A–E). Among the three doxorubicin‐induced experimental groups, the apoptosis rate in 40 nM doxorubicin was the lowest (Figure 4B–E). We also found that the proliferative activity of three doxorubicin‐induced groups was all significantly decreased than that of the control group, and the 40 nM doxorubicin group was the least affected (Figure 4F). These results suggested that less apoptosis phenomenon and a more proliferative phenomenon existed in the higher senescent group. Together, we speculated that senescent lymphoma cells might have a certain antiapoptosis effect and good proliferative activity.

### 3.5 Senescent DLBCL cells performed the unique SASP

In this study, we also tested the level of secreted cytokines in the cell suspension from doxorubicin‐induced senescent LY8 cells. The results showed that compared with the control group, except for VEGF, the other cytokines including of E2F1, IL‐2, IL‐6, IL‐8, IL‐10, IL‐35, and TGF‐β1 were all upregulated in varying degrees of three doxorubicin (10, 20, and 40 nM) induced groups (Figure 4G–N). It was suggested that the stress of doxorubicin‐induced senescent lymphoma cells could increase the levels of various secreted cytokines, which might be one of the key factors to promote the progression of lymphoma.

### 3.6 *SENEX* gene was a key factor controlling SIPS in lymphoma cells

**Figure 5:**
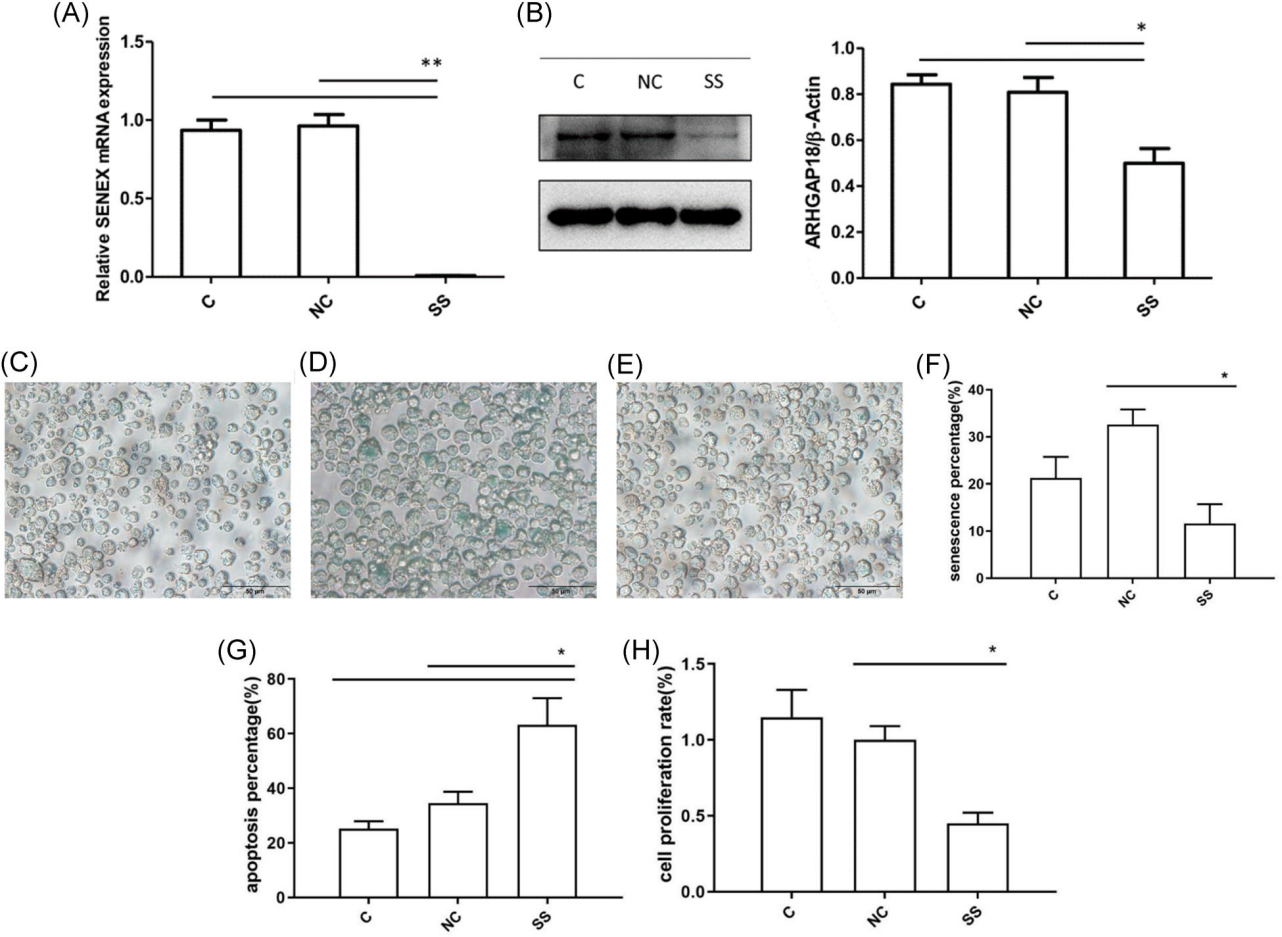
*SENEX* gene was a kted with *SENEX*‐siRNA or NC, and then total RNA and protein were extracted for qRT‐PCR and western blot analysis. (A) The expression of *SENEX* mRNA was measured by qRT‐PCR. (B) The expression of ARHGAP18 was measured by western blot. (C) Unprocessed LY8 cells; NC represented LY8 cells transfected with NC; SS represented LY8 cells transfected with *SENEX*‐siRNA. (C–F) The senescence rate of LY8 cells transfected with *SENEX*‐siRNA. (G) The apoptosis rate of LY8 cells transfected with *SENEX*‐siRNA. (H) The cell proliferation rate of LY8 cells transfected with *SENEX*‐siRNA. **p< .01, *p< .05. mRNA, messenger RNA; NC, negative control; siRNA, small interfering RNA; qRT‐PCR, quantitative real‐time polymerase chain reaction

To further explore the function of the *SENEX* gene in DLBCL, we silenced the *SENEX* gene by RNA interference in LY8 cells (Figure 5A,B) and then used doxorubicin to induce the SIPS model. Interestingly, compared with the control group (C group) and scramble negative control (NC group), the senescence rate and proliferation rate were significantly decreased (Figure 5C–G), but the apoptosis rate was significantly increased in *SENEX* silenced group (SS group) (Figure 5G). These results indicated that the *SENEX* gene was a key factor controlling the formation and apoptosis resistance of SIPS in lymphoma cells. We suggested SIPS induced by the *SENEX* gene mediates apoptosis resistance of DLBCL via promoting immunosuppressive cells and cytokines.

## 4 DISCUSSION

DLBCL is a heterogeneous lymphoid malignancy and the most important subtype of non‐Hodgkin's lymphoma with one of the highest mortality rates. Thirty to 40% of patients will develop r/r disease that remains a major cause of mortality due to the limited therapeutic options.^19^ Recently, studies on DLBCL have focused on the different role of the tumor microenvironment (TME), which is based on the relationship between tumor cells, immune cells, and inflammatory cells.^20^ In contrast to quiescent cells, senescent cells are usually bigger.^21^ SA‐β‐Gal activity is a widely used marker for senescent cells.^22^ In this study, we demonstrated that enlarged, SA‐β‐Gal staining positive lymphoma cells were increased in the peripheral blood and lymph node specimens of r/r DLBCL patients. These outcomes illustrated an important clinical phenomenon in that senescent cells and most likely senescent lymphoma cells were increased in r/r DLBCL.

SIPS is the most important inducer of cellular senescence and has a dual role in developing diseases and response to therapy in vivo.^23^ Recent studies suggest that SIPS may be invoked during the exposure of tumor cells to conventional chemotherapy. In particular, the data show that senescence of tumor cells is a reversible process, treatments that enhance senescence are unlikely to provide a useful therapeutic strategy against the tumor.^24^, ^25^, ^26^ However, whether or not SIPS is present and how does SIPS affects DLBCL remains inconclusive.

In the present study, different concentrations of doxorubicin were applied to LY8 cells and introduced for 2 h each time once every 2 days six times because SA‐β‐Gal expression and the characteristic morphology take at least a few days (usually 3–7 days) after the different treatments in tumor cells.^27^ The proportion of senescent lymphoma cells identified with SA‐β‐Gal staining was increased, and consistent with high expression of p16 and pRB protein. Our results demonstrated that the SIPS of lymphoma cells were successfully induced with doxorubicin in vitro. We next examined the apoptosis rate and proliferative activity in the above LY8 cells within the state of SIPS and found that the senescent lymphoma cells possessed the characteristics of a lower apoptosis rate and higher proliferative activity. Together, these results illustrated that senescent lymphoma cells under the state of SIPS might have antiapoptotic effects and proliferative activity, which might be an important factor contributing to lymphoma cell resistance against drugs and disease progression.

Senescence‐associated gene and genes encoding the secretory proteins changes are mostly specific within individual cell types.^28^ The most significant among the genes and proteins is the acquisition of SASP that is composed of several soluble and insoluble factors. These factors can affect surrounding cells by activating various cell‐surface receptors and the corresponding signal transduction pathways and may lead to multiple pathologies.^12^ One of the most important effects of SASP is to promote the proliferation, invasion, and migration of those cells.^12^, ^28^ SASP is also held responsible for senescence spreading in other cell subtypes.^24^ Interestingly, although a core of SASP factors is a feature of all senescent cells, there are variations of the SASP that depend on the cell type and senescence inducer.^12^ Although many inflammatory factors contribute to the SASP, the inflammatory cytokines, such as IL‐6 and IL‐8, are usually the most common factors.^29^, ^30^, ^31^, ^32^ In this study, our results showed that when the proportion of senescent cells increased, the cytokines in either patient serum or culture supernatant of lymphoma cell line included IL‐2, IL‐6, IL‐8, and IL‐35, IL‐10, TGF‐β1 and TNF‐α were all up‐regulated to varying degrees, which constitutes a unique secretion phenotype of pro‐inflammatory and immunosuppressive. We believe that stress‐induced senescent lymphoma cells increase the levels of various secreted cytokines, forming a unique SASP microenvironment. SASP may interact and participate in the formation of complex TME, and lead to the anti‐tumor and proliferative activities of senescent lymphoma cells.

Treg and MDSC are the other cellular components of TME that can contribute to regulating the antitumor immune responses by suppressing T cells proliferation in DLBCL.^33^, ^34^ Our results showed that Treg and MDSC including M‐MDSC and G‐MDSC were both upregulated in the peripheral blood of r/r DLBCL patients. The accumulation of these immunosuppressive cells might be one of the important partners of SASP that collaboratively caused lymphoma cells' immune escape and drug resistance.

Coleman et al. first reported that the *SENEX* gene has been confirmed to have a unique gatekeeper function in the SIPS and apoptosis pathways in endothelial cells. The *SENEX* induced senescent endothelial cells show anti‐inflammatory properties and have an enhanced barrier function to protect against apoptosis.^13^ In aged bladder cancer patients, our previous study has shown that *SENEX* gene expression was upregulated in Treg in response to H_2_O_2_‐mediated stress, but silencing it could increase Tregs' apoptosis and promote proapoptotic gene expression.^35^ Recently, we found that SIPS can be induced by H_2_O_2_ in DLBCL cells. SIPS formation promotes the expression of the *SENEX* gene and the p16/Rb pathway in DLBLC cells, while proliferation can be investigated in the above cells.^16^ In the present study, we found that SIPS promoted apoptosis resistance of DLBCL via increasing immunosuppressive cells and SASP, and this process was regulated by the senescence gene *SENEX*.

There are still some limitations to this study. First, due to the limited clinical cases, we did not further compare senescent cells and immunosuppressive components in patients with different stages and types. Next, we only used the LY8 cell in vitro experiment in the study, this lymphoma cell line is derived from germinal cancer and doesn't represent a nongerminal center source of DLBCL. Therefore, further research is needed to testify and validate the finding of our study.

In conclusion, it has been reported that resistance to apoptosis is associated with the progression of DLBCL, but the mechanism is unknown. Our results herein illustrated SIPS activated by the *SENEX* gene mediates apoptosis resistance of r/r DLBCL via promoting immunosuppressive cells and SASP. The definite mechanism of SIPS at the molecular gene level should be further investigated.

## CONFLICT OF INTERESTS

The authors declare that there are no conflict of interests.

## AUTHOR CONTRIBUTIONS

*Designed the experiment*: Zhimin Zhai. *Performed all the experiments*: Jiyu Wang, Zhixiang Wanyan, and Fengfeng Zhu; *Wrote the manuscript*: Qianshan Tao and Jiyu Wang; *Revised the manuscript*: Ying Pan; *Analyzed the data*: Jiyu Wang and Qianshan Tao; *Collected the clinical specimens*: Ying Pan, Huiping Wang, Xuanxuan Xu; LY and MZ *Contributed to clinical data acquisition*: Liuying Yi and Mei Zhou. All authors have read and agreed to the published version of the manuscript.

## Data Availability

The data used in this article are available from the corresponding author upon request

## References

[1] Miao Y , Medeiros LJ , Xu‐Monette ZY , Li J , Young KH Dysregulation of cell survival in diffuse large B cell lymphoma: mechanisms and therapeutic targets. Front Oncol. 2019;9:107. 30881917 10.3389/fonc.2019.00107 PMC6406015

[2] Raut LS , Chakrabarti PP Management of relapsed‐refractory diffuse large B cell lymphoma. South Asian J Cancer. 2014;3(1):66‐70. 24665451 10.4103/2278-330X.126531 PMC3961873

[3] Gisselbrecht C , Glass B , Mounier N , et al. Salvage regimens with autologous transplantation for relapsed large B‐cell lymphoma in the rituximab era. J Clin Oncol. 2010;28(27):4184‐4190. 20660832 10.1200/JCO.2010.28.1618 PMC3664033

[4] Batlevi CL , Matsuki E , Brentjens RJ , Younes A Novel immunotherapies in lymphoid malignancies. Nat Rev Clin Oncol. 2016;13(1):25‐40. 26525683 10.1038/nrclinonc.2015.187 PMC4916838

[5] Hayflick L , Moorhead PS The serial cultivation of human diploid cell strains. Exp Cell Res. 1961;25:585‐621. 13905658 10.1016/0014-4827(61)90192-6

[6] von Kobbe C Cellular senescence: a view throughout organismal life. Cell Mol Life Sci. 2018;75(19):3553‐3567. 30030594 10.1007/s00018-018-2879-8 PMC11105332

[7] Bernadotte A , Mikhelson VM , Spivak IM . Markers of cellular senescence. Telomere shortening as a marker of cellular senescence. Aging (Albany NY). 2016;8(1):3‐11. 26805432 10.18632/aging.100871 PMC4761709

[8] Goligorsky MS , Hirschi K Stress‐induced premature senescence of endothelial and endothelial progenitor cells. Adv Pharmacol. 2016;77:281‐306. 27451101 10.1016/bs.apha.2016.04.007 PMC5206791

[9] Collado M , Blasco MA , Serrano M . Cellular senescence in cancer and aging. Cell. 2007;130(2):223‐233. 17662938 10.1016/j.cell.2007.07.003

[10] Oshima J , Sidorova JM , Monnat RJ, Jr. Werner syndrome: clinical features, pathogenesis and potential therapeutic interventions. Ageing Res Rev. 2017;33:105‐114. 26993153 10.1016/j.arr.2016.03.002 PMC5025328

[11] Ruhland MK , Loza AJ , Capietto AH , et al. Stromal senescence establishes an immunosuppressive microenvironment that drives tumorigenesis. Nat Commun. 2016;7:11762. 27272654 10.1038/ncomms11762 PMC4899869

[12] Coppe JP , Desprez PY , Krtolica A , Campisi J The senescence‐associated secretory phenotype: the dark side of tumor suppression. Annu Rev Pathol. 2010;5:99‐118. 20078217 10.1146/annurev-pathol-121808-102144 PMC4166495

[13] Coleman PR , Hahn CN , Grimshaw M , et al. Stress‐induced premature senescence mediated by a novel gene, SENEX, results in an anti‐inflammatory phenotype in endothelial cells. Blood. 2010;116(19):4016‐4024. 20664062 10.1182/blood-2009-11-252700

[14] Humphries B , Wang Z , Li Y , Jhan JR , Jiang Y , Yang C ARHGAP18 downregulation by miR‐200b suppresses metastasis of triple‐negative breast cancer by enhancing activation of RhoA. Cancer Res. 2017;77(15):4051‐4064. 28619708 10.1158/0008-5472.CAN-16-3141

[15] Li Y , Ji S , Fu L , Jiang T , Wu D , Meng F Over‐expression of ARHGAP18 suppressed cell proliferation, migration, invasion, and tumor growth in gastric cancer by restraining over‐activation of MAPK signaling pathways. Onco Targets Ther. 2018;11:279‐290. 29386906 10.2147/OTT.S130255 PMC5767098

[16] Wang J , Wang Z , Wang H , et al. Stress‐induced premature senescence promotes proliferation by activating the SENEX and p16(INK4a)/retinoblastoma (Rb) pathway in diffuse large B‐cell lymphoma. Turk J Haematol. 2019;36(4):247‐254. 31327185 10.4274/tjh.galenos.2019.2019.0117 PMC6863019

[17] Xue Zhonghua , Xue Ye , Zhi Za . Chinese guidelines for diagnosis and treatment of malignant lymphoma 2018. Chin J Cancer Res. 2013;34(9):816‐819.

[18] Wang JY , Fang M , Boye A , et al. Interaction of microRNA‐21/145 and Smad3 domain‐specific phosphorylation in hepatocellular carcinoma. Oncotarget. 2017;8(49):84958‐84973. 29156696 10.18632/oncotarget.17709 PMC5689586

[19] Teras LR , DeSantis CE , Cerhan JR , Morton LM , Jemal A , Flowers CR US lymphoid malignancy statistics by World Health Organization subtypes. CA Cancer J Clin. 2016;66(6):443‐459. 27618563 10.3322/caac.21357

[20] Cioroianu AI , Stinga PI , Sticlaru L , et al. Tumor microenvironment in diffuse large B‐cell lymphoma: role and prognosis. Anal Cell Pathol (Amst). 2019;2019:8586354‐8586359. 31934533 10.1155/2019/8586354 PMC6942707

[21] Ksiazek K , Korybalska K , Jorres A , Witowski J . Accelerated senescence of human peritoneal mesothelial cells exposed to high glucose: the role of TGF‐beta1. Lab Invest. 2007;87(4):345‐356. 17297436 10.1038/labinvest.3700519

[22] Dimri GP , Lee X , Basile G , et al. A biomarker that identifies senescent human cells in culture and in aging skin in vivo. Proc Natl Acad Sci USA. 1995;92(20):9363‐9367. 7568133 10.1073/pnas.92.20.9363 PMC40985

[23] Schosserer M , Grillari J , Breitenbach M The dual role of cellular senescence in developing tumors and their response to cancer therapy. Front Oncol. 2017;7:278. 29218300 10.3389/fonc.2017.00278 PMC5703792

[24] Mikula‐Pietrasik J , Niklas A , Uruski P , Tykarski A , Ksiazek K Mechanisms and significance of therapy‐induced and spontaneous senescence of cancer cells. Cell Mol Life Sci. 2020;77(2):213‐229. 31414165 10.1007/s00018-019-03261-8 PMC6970957

[25] Sun Y , Coppe JP , Lam EW Cellular senescence: the sought or the unwanted? Trends Mol Med. 2018;24(10):871‐885. 30153969 10.1016/j.molmed.2018.08.002

[26] Mavrogonatou E , Pratsinis H , Kletsas D . The role of senescence in cancer development. Semin Cancer Biol. 2019;62:182‐191. 31260734 10.1016/j.semcancer.2019.06.018

[27] Ewald JA , Desotelle JA , Wilding G , Jarrard DF Therapy‐induced senescence in cancer. J Natl Cancer Inst. 2010;102(20):1536‐1546. 20858887 10.1093/jnci/djq364 PMC2957429

[28] Faget DV , Ren Q , Stewart SA Unmasking senescence: context‐dependent effects of SASP in cancer. Nat Rev Cancer. 2019;19(8):439‐453. 31235879 10.1038/s41568-019-0156-2

[29] Demaria M , O'Leary MN , Chang J , et al. Cellular senescence promotes adverse effects of chemotherapy and cancer relapse. Cancer Discov. 2017;7(2):165‐176. 27979832 10.1158/2159-8290.CD-16-0241 PMC5296251

[30] Malaponte G , Hafsi S , Polesel J , et al. Tumor microenvironment in diffuse large B‐cell lymphoma: matrixmetalloproteinases activation is mediated by osteopontin overexpression. Biochim Biophys Acta. 2016;1863(3):483‐489. 26381542 10.1016/j.bbamcr.2015.09.018

[31] Nacinovic‐Duletic A , Stifter S , Dvornik S , Skunca Z , Jonjic N Correlation of serum IL‐6, IL‐8 and IL‐10 levels with clinicopathological features and prognosis in patients with diffuse large B‐cell lymphoma. Int J Lab Hematol. 2008;30(3):230‐239. 18479302 10.1111/j.1751-553X.2007.00951.x

[32] Manfroi B , McKee T , Mayol JF , et al. CXCL‐8/IL8 produced by diffuse large B‐cell lymphomas Recruits neutrophils expressing a proliferation‐inducing ligand APRIL. Cancer Res. 2017;77(5):1097‐1107. 27923834 10.1158/0008-5472.CAN-16-0786

[33] Gomez‐Gelvez JC , Salama ME , Perkins SL , Leavitt M , Inamdar KV . Prognostic impact of tumor microenvironment in diffuse large B‐cell lymphoma uniformly treated with R‐CHOP chemotherapy. Am J Clin Pathol. 2016;145(4):514‐523. 27124945 10.1093/ajcp/aqw034

[34] Marvel D , Gabrilovich DI Myeloid‐derived suppressor cells in the tumor microenvironment: expect the unexpected. J Clin Invest. 2015;125(9):3356‐3364. 26168215 10.1172/JCI80005 PMC4588239

[35] Chen T , Wang H , Zhang Z , et al. A novel cellular senescence gene, SENEX, is involved in peripheral regulatory T cells accumulation in aged urinary bladder cancer. PLOS One. 2014;9(2):e87774. 24505313 10.1371/journal.pone.0087774 PMC3914842

